# Development of a multisystem point of care ultrasound skills assessment checklist

**DOI:** 10.1186/s13089-022-00268-4

**Published:** 2022-05-12

**Authors:** Nilam J. Soni, Robert Nathanson, Mark Andreae, Rahul Khosla, Karthik Vadamalai, Karthik Kode, Jeremy S. Boyd, Charles M. LoPresti, Dana Resop, Zahir Basrai, Jason Williams, Brian Bales, Harald Sauthoff, Erin Wetherbee, Elizabeth K. Haro, Natalie Smith, Michael J. Mader, Jacqueline Pugh, Erin P. Finley, Christopher K. Schott

**Affiliations:** 1 Medicine Service, South Texas Veterans Health Care System, San Antonio, TX USA; 2 Department of Medicine, University of Texas Health San Antonio, 7703 Floyd Curl Drive, MC 7982, San Antonio, TX 78229 USA; 3 Departments of Critical Care Medicine and Emergency Medicine, University of Pittsburgh and University of Pittsburgh Medical Center, Pittsburgh, PA USA; 4 Veterans Affairs Medical Center, Washington, DC USA; 5 George Washington University, Washington, DC USA; 6 Critical Care Medicine, Mercy Hospital, Springfield, MO USA; 7 Department of Medicine, University of Hawai’i at Manoa John A. Burns School of Medicine, Honolulu, HI USA; 8 Emergency Medicine, Veterans Affairs Tennessee Valley Healthcare System, Nashville, TN USA; 9 Department of Emergency Medicine, Vanderbilt University Medical Center, Nashville, TN USA; 10 Medicine Service, Louis Stokes Cleveland Veterans Affairs Medical Center, Cleveland, OH USA; 11 Department of Medicine, Case Western Reserve University School of Medicine, Cleveland, OH USA; 12 Department of Emergency Medicine, University of Wisconsin, Madison, WI USA; 13 Emergency Department, William S. Middleton Memorial Veterans Hospital, Madison, WI USA; 14 Emergency Medicine, VA Greater Los Angeles Healthcare System, Los Angeles, CA USA; 15 Section of Hospital Medicine, Atlanta Veterans Affairs Medical Center, Atlanta, GA USA; 16 Division of Hospital Medicine, Emory School of Medicine, Atlanta, GA USA; 17 Medicine Service, Veterans Affairs New York Harbor Healthcare System, New York, NY USA; 18 Division of Pulmonary, Critical Care, and Sleep Medicine, New York University Grossman School of Medicine, New York, NY USA; 19 Division of Pulmonary, Critical Care, and Sleep Medicine, University of Minnesota, Minneapolis, MN USA; 20 Pulmonary, Critical Care, and Sleep Apnea, Minneapolis Veterans Affairs Health Care System, Minneapolis, MN USA; 21 Research Service, South Texas Veterans Health Care System, San Antonio, TX USA; 22 Department of Critical Care Medicine, Veterans Affairs of Pittsburgh Health Care Systems, Pittsburgh, PA USA

**Keywords:** Education, Point of care, Ultrasound, Checklist

## Abstract

**Background:**

Many institutions are training clinicians in point-of-care ultrasound (POCUS), but few POCUS skills checklists have been developed and validated. We developed a consensus-based multispecialty POCUS skills checklist with anchoring references for basic cardiac, lung, abdominal, and vascular ultrasound, and peripheral intravenous line (PIV) insertion.

**Methods:**

A POCUS expert panel of 14 physicians specializing in emergency, critical care, and internal/hospital medicine participated in a modified-Delphi approach to develop a basic POCUS skills checklist by group consensus. Three rounds of voting were conducted, and consensus was defined by ≥ 80% agreement. Items achieving < 80% consensus were discussed and considered for up to two additional rounds of voting.

**Results:**

Thirteen POCUS experts (93%) completed all three rounds of voting. Cardiac, lung, abdominal, and vascular ultrasound checklists included probe location and control, basic machine setup, image quality and optimization, and identification of anatomical structures. PIV insertion included additional items for needle tip tracking. During the first round of voting, 136 (82%) items achieved consensus, and after revision and revoting, an additional 21 items achieved consensus. A total of 153 (92%) items were included in the final checklist.

**Conclusions:**

We have developed a consensus-based, multispecialty POCUS checklist to evaluate skills in image acquisition and anatomy identification for basic cardiac, lung, abdominal, and vascular ultrasound, and PIV insertion.

**Supplementary Information:**

The online version contains supplementary material available at 10.1186/s13089-022-00268-4.

## Background

Point-of-care ultrasound (POCUS) training is required for a growing list of specialties, including emergency medicine, critical care, and anesthesiology [1]. Physicians in-practice have been obtaining training through local and national continuing medical education courses that provide hands-on training and have been shown to be effective [2, 3].

Despite an increase in POCUS training, a critical gap remains in the ability to determine a physician’s competency in POCUS use due to variability in training standards and definitions of competency [4]. Several checklists and global rating scales have been published to evaluate POCUS skills [5–14]. Most published checklists are limited to a single organ system or specialty, and no multispecialty, multisystem checklists for evaluation of common POCUS applications of the lungs, heart, abdomen, and lower extremity veins have been published. Hospitals and healthcare systems are seeking validated multisystem POCUS checklists that can be applied across specialties to certify physician skills and maintain standards for POCUS use.

We describe the development of a multispecialty, multisystem POCUS skills checklist based on group consensus of national POCUS faculty from distinct institutions as the initial step toward creating a validated checklist.

## Methods

We conducted a prospective observational study using consensus-based methods in two phases. The University of Pittsburgh Institutional Review Board approved this project (IRB # PRO18050302).

The initial POCUS skills checklist was developed by group consensus of POCUS experts from emergency medicine, critical care medicine, and hospital medicine during an in-person 3-day meeting dedicated toward developing a national POCUS training course for physicians practicing in the Department of Veterans Affairs (VA). Three diagnostic (heart, lungs, and abdomen) and one procedural application [peripheral intravenous line (PIV) insertion] were included in the checklist based on current evidence and applicability to multiple specialties. We piloted the initial checklist to evaluate novice learners from 2017 to 2019. Based on faculty feedback, the initial checklist was revised to include one additional diagnostic application (lower extremity deep venous thrombosis).

To gather formal consensus, an expert panel of 14 national POCUS faculty from emergency, critical care, and hospital medicine was convened which included the experts that developed the initial POCUS skills checklist. Experts were defined as individuals who regularly used POCUS in clinical practice; taught POCUS courses locally or nationally; and either had completed a dedicated POCUS fellowship, had a national professional society leadership role in POCUS, or had previously published on POCUS topics. All experts were required to disclose any conflicts of interest.

The checklist was divided into five sections (cardiac, lung, abdomen, lower extremity DVT, and PIV) and entered in an internet-based electronic data collection instrument (Research Electronic Data Capture [REDCap™]) hosted on the server of the University of Texas Health Science Center in San Antonio, Texas. Expert panel members rated each item as a requirement for basic competency, and panel members were encouraged to provide feedback in free text boxes for each item.

A modified-Delphi approach was used to assess the level of agreement among experts. Three rounds of electronic voting followed by group discussion by videoconferencing were conducted between May 2020 and December 2020. Consensus was defined by ≥ 80% of experts agreeing to include an item. Items achieving < 80% consensus for inclusion were discussed, revised, and considered for an additional two rounds of voting.

To finalize the checklist, we pilot tested it on pre-recorded skill examinations of 18 learners who were categorized as novice, intermediate, or experienced POCUS users based on learners’ prior training and current use. Each POCUS expert reviewed a minimum of 40 videos that were randomized by learner and expert reviewer, and each video was rated by at least five different experts. Feedback from raters was incorporated into the checklist to add anchors and clarify wording. Formal validation of the checklist is planned for the future when the COVID-19 pandemic subsides and live in-person POCUS training events are permitted.

## Results

Fourteen POCUS experts participated and 13 (93%) completed all three rounds of voting. Characteristics of the POCUS expert panel are displayed in Additional file 1: Table S1.

The original skills checklist included a total of 166 items for five different POCUS applications. The cardiac, lung, abdominal, and lower extremity DVT ultrasound checklists included sections for probe type, location, and control; basic machine setup; image quality and optimization; and identification of anatomical structures. The checklist for PIV insertion included additional items for needle-tip tracking.

After the first round of voting, 136 (82%) checklist items achieved consensus based on ≥ 80% agreement for inclusion (Additional file 2: Table S2). Thirty items did not achieve consensus from the cardiac (17), lung (6), PIV insertion (3), abdomen (2), and lower extremity DVT (2) checklists. A checklist item for speed and efficiency consistently did not achieve consensus for all applications and was removed after the follow-up panel discussion.

The second round of voting included 19 checklist items. Prior to voting, checklist items for optimization of image depth and gain were revised as, “Image depth (or gain) optimized appropriately.” Differences in convention of the screen marker and image orientation for the subcostal 4-chamber and inferior vena cava views were discussed and clarified on the checklist during the second round of voting. We chose to allow some flexibility and stated, “exam preset and orientation can vary based on specialty or local convention.” An additional 15 items reached consensus after the second round of voting.

The third round of voting focused on cardiac subcostal views. In emergency medicine, the subcostal views are often obtained as part of a focused assessment with sonography in trauma (FAST) exam using a curvilinear probe and an abdominal exam preset, whereas in internal medicine and critical care, these views are most often obtained as part of a cardiac evaluation using a phased-array probe and cardiac exam preset. The group felt strongly to not remove these items and both probe types and exam settings were included in the revised checklist. All three items in the third round of voting achieved consensus. A total of 153 items were included in the final checklist (Tables 1, 2, 3 and 4).

**Table 1:** Cardiac POCUS checklist for basic competency in image acquisition and anatomy identification

Cardiac parasternal	Cardiac subxiphoid
Selects PHASED-ARRAY transducer	Selects PHASED-ARRAY transducer
Selects CARDIAC EXAM	Selects CARDIAC EXAM
Correct probe LOCATION	Correct Probe LOCATION
Correct probe ORIENTATION	Correct Probe ORIENTATION
Probe CONTROL	Probe CONTROL
Obtains a quality Parasternal Long Axis view *(Required structures* = *RV, LV, LA, AV, MV, LVOT, aortic root, descending thoracic aorta)*	Obtains quality subxiphoid 4-chamber VIEW *(Required structures* = *Liver, RV, RA, TV, LV, LA, MV)*
Points to RIGHT VENTRICLE	Points to PERICARDIUM
Points to LEFT VENTRICLE	Points to LIVER
Points to LEFT ATRIUM	Points to RIGHT VENTRICLE
Points to AORTIC VALVE	Points to RIGHT ATRIUM
Points to MITRAL VALVE	Points to LEFT VENTRICLE
Points to LEFT VENTRICULAR OUTFLOW TRACK	Points to LEFT ATRIUM
Points to DESCENDING THORACIC AORTA	Points to MITRAL VALVE
Points to PERICARDIUM	Points to TRICUSPID VALVE
Image DEPTH optimized appropriately	Image DEPTH optimized appropriately
Image GAIN optimized appropriately	Image GAIN optimized appropriately

Cardiac apical	Inferior vena cava
Selects PHASED-ARRAY transducer	Selects PHASED-ARRAY or CURVILINEAR transducer
Selects CARDIAC EXAM	Selects CARDIAC or ABDOMINAL EXAM preset
Correct Probe LOCATION	Correct Probe LOCATION
Correct Probe ORIENTATION	Correct Probe ORIENTATION
Probe CONTROL	Probe CONTROL
Obtains a quality 4-chamber cardiac VIEW (a 5-chamber view is acceptable) *(Required structures* = *RV, LV, RA, LA, TV, MV)*	Obtains quality IVC VIEW *(Required structures* = *RA, IVC, a hepatic vein, liver)*
Points to RIGHT VENTRICLE	Points to LIVER
Points to LEFT VENTRICLE	Points to IVC
Points to RIGHT ATRIUM	Points to HEPATIC VEIN
Points to LEFT ATRIUM	Points to RIGHT ATRIUM
Points to MITRAL VALVE	Points to site to assess for RESPIRATORY VARIATION
Points to TRICUSPID VALVE	–
Image DEPTH optimized appropriately	Image DEPTH optimized appropriately
Image GAIN optimized appropriately	Image GAIN optimized appropriately
**Based on the overall performance of this learner through all the views obtained and identification of anatomic structures during this hands-on skills evaluation, do you consider this learner to have the minimum skills to be considered COMPETENT** **in image acquisition and anatomy identification to perform CARDIAC** **POCUS exams of patients?**	

*AV* aortic valve, *IVC* inferior vena cava, *LA* left atrium, *LV* left ventricle, *LVOT* left ventricular outflow tract, *MV* mitral valve, *POCUS* point-of-care ultrasound, *RA* right atrium, *RV* right ventricle, *TV* tricuspid valve

**Table 2:** Lung POCUS checklist for basic competency in image acquisition and anatomy identification

Lung—anterior chest	Lung—costophrenic recess
Selects phased-array or curvilinear probe	Selects phased-array or curvilinear probe
Selects ABDOMINAL or LUNG EXAM	Selects ABDOMINAL or LUNG EXAM
Correct Probe LOCATION	Correct Probe LOCATION
Correct Probe ORIENTATION	Correct Probe ORIENTATION
Probe CONTROL	Probe CONTROL
Obtains quality Anterior Lung VIEW *(Required structures* = *Ribs, pleural line, A-lines)*	Obtains quality Costophrenic VIEW *(Required structures* = *Diaphragm, liver or spleen, lung parenchyma descending with respirations)*
Points to RIBS	Points to LIVER or SPLEEN
Points to RIB SHADOW	Points to DIAPHRAGM
Points to PLEURAL LINE	Points to LUNG PARENCHYMA
Recognizes PLEURAL SLIDING	Points to COSTOPHRENIC RECESS
Points to A-LINES	–
Demonstrates normal pattern using M-mode (seashore)	–
Image DEPTH optimized appropriately	Image DEPTH optimized appropriately
Image GAIN optimized appropriately	Image GAIN optimized appropriately
**Based on the overall performance of this learner through all the views obtained and identification of anatomic structures during this hands-on skills evaluation, do you consider this learner to have the minimum skills to be considered** **COMPETENT** **in image acquisition and anatomy identification to perform LUNG and PLEURAL** POCUS exams of patients?

**Table 3:** Abdominal and pelvic POCUS checklists for basic competency in image acquisition and anatomy identification

Abdominal skills	Pelvic skills
Selects curvilinear or phased-array probe	Selects curvilinear or phased-array probe
Selects ABDOMINAL EXAM	Selects ABDOMINAL EXAM
Correct Probe LOCATION	Correct Probe LOCATION
Correct Probe ORIENTATION	Correct Probe ORIENTATION
Probe CONTROL	Probe CONTROL
Obtains quality right upper quadrant FAST VIEW *(Required structures* = *liver, kidney (including inferior pole of kidney), hepatorenal recess)*	Obtains quality transverse BLADDER VIEW with either the prostate or uterus in view *(Required structures* = *bladder, prostate/uterus)*
Points to DIAPHRAGM	Points to BLADDER WALL
Points to LIVER	Points to PROSTATE or UTERUS
Points to KIDNEY	Points to URINE WITHIN THE BLADDER
Points to HEPATORENAL RECESS (Morison’s Pouch)	Points to area to assess for PELVIC FREE FLUID
Points to RENAL PELVIS	–
Image DEPTH optimized appropriately	Image DEPTH optimized appropriately
Image GAIN optimized appropriately	Image GAIN optimized appropriately
**Based on the overall performance of this learner through all the views obtained and identification of anatomic structures during this hands-on skills evaluation, do you consider this learner to have the minimum skills to be considered COMPETENT** **in image acquisition and anatomy identification to perform ABDOMINAL and PELVIC** **POCUS exams of patients?**

**Table 4:** Vascular POCUS checklist for basic competency in image acquisition and anatomy identification

Lower extremity deep venous thrombosis (DVT)	Peripheral IV insertion (transverse approach)
Selects high freq linear transducer	Selects high freq linear transducer
Selects vascular, venous, or arterial EXAM	Select vascular, venous, or arterial EXAM
Correct probe LOCATION	Correct probe LOCATION
Correct probe ORIENTATION	Correct probe ORIENTATION
Probe CONTROL	Probe CONTROL
Obtains quality COMMON FEMORAL VEIN VIEW *(Vein should appear oval/triangular in the center of screen; avoid oblique, off-axis views)*	Tracks needle tip as needle advances toward vein Successfully uses transverse approach to insert peripheral IV
Points to common femoral vein Points to common femoral artery Points to common femoral vein/greater saphenous vein junction Demonstrates common femoral vein COMPRESSION Points to femoral vein Demonstrates femoral vein COMPRESSION	Image DEPTH optimized appropriately Image GAIN optimized appropriately
	**Peripheral IV insertion (longitudinal approach)**
	Selects high freq linear transducer Selects Vascular, Venous, or Arterial EXAM Correct Probe LOCATION Correct Probe ORIENTATION Probe CONTROL
Obtains quality POPLITEAL VEIN VIEW *(Both popliteal vein and artery should be seen clearly in center of screen)*	Tracks needle tip as needle advances toward target vessel in longitudinal orientation Successfully uses longitudinal approach to insert PIV
Points to popliteal artery	Image DEPTH optimized appropriately Image GAIN optimized appropriately
Points to popliteal vein	
Demonstrates popliteal venous COMPRESSION	
Image DEPTH optimized appropriately Image GAIN optimized appropriately	–
**Based on the overall performance of this learner through all the views obtained and identification of anatomic structures during this hands-on skills evaluation, do you consider this learner to have the minimum skills to be considered COMPETENT** **in image acquisition and anatomy identification to perform LOWER EXTREMITY DVT** **POCUS exams of patients?**	**Based on the overall performance of this learner through all the views obtained and identification of anatomic structures during this hands-on skills evaluation, do you consider this learner to have the minimum skills to be considered COMPETENT** **to perform ultrasound-guided PERIPHERAL IV INSERTION** **on patients?**

*IV* intravenous

The checklist was pilot tested using pre-recorded videos to identify unclear or ambiguous checklist items that could have varying interpretations. Anchors and explanatory statements were added to clarify certain checklist items based on group discussion (Additional file 3: Table S3).

## Discussion

We have developed a consensus-based multisystem POCUS skills checklist to assess basic competency in image acquisition and anatomy identification. The checklist includes 153 items to evaluate skills to perform basic cardiac, lung, abdominal, and vascular ultrasound applications, including PIV insertion, that are commonly used in emergency medicine, critical care, and hospital medicine.

Our POCUS skills assessment checklist has noteworthy differences from other checklists. Most published POCUS skills checklists focus on assessing image acquisition skills of a single organ system, such as cardiac [5–8], thoracic [12–14], FAST exam [9–11], vascular [5], neuromuscular [15], musculoskeletal [16], or procedures [17]; or assessing skills of clinicians from a single specialty, such as emergency medicine [10], surgery [9], or critical care [5]. In contrast, our checklist was based on consensus from 14 POCUS experts from emergency (5), critical care (5), and hospital medicine (4), who practice at different medical centers across the United States. The value of our consolidated checklist is it establishes a common standard for assessing skills in image acquisition and anatomy identification for basic, common POCUS applications across specialties. Institutions seeking tools to assess POCUS skills prior to granting privileges to use POCUS for clinical decision-making can use our checklist to efficiently evaluate POCUS skills of physicians from different specialties.

Our multisystem POCUS skills checklist combines the use of both checklist items and global rating scales. Checklists use task-specific items that can provide both evaluative scoring with cutoff levels for “passing” as well as formative feedback. Checklists are perceived as being easier to use, especially for non-expert assessors, and having better interrater reliability [17]. However, checklists may focus more on thoroughness rather than overall competency and may not capture a summative assessment of one’s performance [18, 19]. One approach to overcome this limitation is increasing the point-value of critical checklist items, or identifying checklist items that result in immediate disqualification from competency if performed incorrectly [18, 20]. By comparison, global rating scales provide an overall assessment of a learner’s skills and can differentiate learner levels with high reliability and sensitivity, particularly when performed by content experts [21–23]. For these reasons, a final global rating question was included to determine whether the learner has demonstrated minimum skills to be considered competent in image acquisition and anatomy identification to perform the specified POCUS exam on patients.

A rigorous multi-step process was conducted to develop our checklist from 2017 to 2021. Initially, speed and efficiency of image acquisition were included in the checklist. However, after pilot testing the initial version of our checklist with novices, we noted substantial variability in interpretation and application of these checklist items among expert faculty and removed them, because consensus could not be achieved on the specific wording, anchoring, and scoring of these items. In the final phase of checklist development, a standardized set of recorded skills exams of novice, experienced, and expert learners were reviewed and scored by the expert panel members independently which led to insertion of additional anchors to clarify some checklist items.

Our consensus-based multisystem checklist has limitations. First, POCUS competency requires mastery of image acquisition and interpretation, and integration of findings into clinical decision-making, which include the cognitive, psychomotor, and affective domains of learning [24, 25]. Our POCUS checklist assesses image acquisition skills and identification of normal structures, while additional assessment is needed for the cognitive domain. Second, we were unable to assess interrater reliability of our checklist due to the cancellation of live in-person courses during the COVID-19 pandemic. We plan to validate our checklist with learners after resumption of live in-person POCUS courses in the future. Third, we had to balance completeness versus efficiency when selecting views to include in a multisystem POCUS skills checklist, and although important, certain views, such as the left upper quadrant, were not included based on group consensus. Finally, we have postponed weighting of critical checklist items until validation of our checklist prospectively. We anticipate greater weighting of the final global rating question on competency for granting privileges.

## Conclusions

We have developed a consensus-based multispecialty, multisystem POCUS checklist to assess basic competency in image acquisition and anatomy identification of cardiac, lung, abdominal, and vascular ultrasound, and PIV insertion. This checklist was designed to assess the skills of novice POCUS users from a wide range of specialties. Future steps include validating our checklist with learners during live in-person POCUS courses and determining its interrater reliability.

## Supplementary Information

**Additional file 1: Table S1.** Characteristics of the Point-of-care Ultrasound Expert Panel.

**Additional file 2: Table S2.** Voting Results of Point-of-care Ultrasound Expert Panel.

**Additional file 3: Table S3.** Multisystem Point-of-care Ultrasound Checklist for Basic Competency in Image Acquisition and Anatomy Identification. A total of 153 checklist items reached consensus and include four diagnostic applications (cardiac, lung, abdominal, and vascular ultrasound) and one procedural application (peripheral intravenous line insertion).

## Data Availability

Data is available upon request.

## Abbreviations

ACGME: Accreditation Council for Graduate Medical Education
DVT: Deep vein thrombosis
FAST: Focused assessment with sonography in trauma
PIV: Peripheral intravenous line
POCUS: Point-of-care ultrasound
