# Supplementary material for: Development of a multisystem point of care ultrasound skills assessment checklist
Source: Ultrasound J. 2022 May 12;14:17. doi: 10.1186/s13089-022-00268-4 (PMC9096739; doi:10.1186/s13089-022-00268-4)
Supplement: Supplementary file 1 — Additional file 1: Table S1. Characteristics of the Point-of-care Ultrasound Expert Panel. [file 13089_2022_268_MOESM1_ESM.docx]

**Table S1: Characteristics of the Point-of-care Ultrasound Expert Panel**

| **Characteristic** | **All Experts (%)**  n=14 |
| --- | --- |
| Specialty  Internal Medicine/Hospital Medicine  Emergency Medicine  Pulmonary Medicine and Critical Care Medicine  Emergency Medicine and Critical Care Medicine  Internal Medicine and Critical Care Medicine | 4 (29)  4 (29)  2 (14)  2 (14)  2 (14) |
| Female | 2 (14) |
| United States Region  Northeast (NY, PA)  South (GA, TX, TN)  Midwest (MO, OH, MN, WI)  West (CA, OR, WA, HI) | 3 (21)  5 (36)  4 (29)  2 (14) |
| Completed Ultrasound Fellowship  Yes | 7 (50) |
| Certificate in POCUS (Chest, SHM, APCA)  Yes | 6 (43) |
| Experience Using Ultrasound in Clinical Practice  0-4 years  5-8 years  >8 years | 0 (0)  4 (29)  10 (71) |
| Experience Teaching Point-of-care Ultrasound  0-4 years  5-8 years  >8 years | 2 (14)  7 (50)  5 (36) |
| Years Assessing Ultrasound Skills of Learners  0-4 years  5-8 years  >8 years | 4 (29)  7 (50)  3 (21) |
| Ultrasound-related Peer-reviewed Publications  0-5  6-10  >10 | 10 (71)  1 (7)  3 (21) |
| Voting  Voted in all 3 rounds | 14 (100) |
