# Supplementary material for: Development of a multisystem point of care ultrasound skills assessment checklist
Source: Ultrasound J. 2022 May 12;14:17. doi: 10.1186/s13089-022-00268-4 (PMC9096739; doi:10.1186/s13089-022-00268-4)
Supplement: Supplementary file 2 — Additional file 2: Table S2. Voting Results of Point-of-care Ultrasound Expert Panel. [file 13089_2022_268_MOESM2_ESM.docx]

| **CARDIAC** | | | | | | | |
| --- | --- | --- | --- | --- | --- | --- | --- |
|  | | **Round 1** | | **Round 2** | | **Round 3** | |
| **Proposed Checklist Item** | **# of Voters** | **Required for**  **Competency?** | **Checklist**  **Status** | **Required for**  **Competency?** | **Checklist**  **Status** | **Required for**  **Competency?** | **Checklist**  **Status** |
| ***POCUS Cardiac Parasternal Skills Test*** | | | | | | | |
| Correct probe LOCATION? | 14 | 14 (100%) | Required |  |  |  |  |
| Correct probe ORIENTATION? | 14 | 14 (100%) | Required |  |  |  |  |
| Probe CONTROL? | 14 | 14 (100%) | Required |  |  |  |  |
| Speed & EFFICIENCY | 14 | 8 (57%) | No |  |  |  |  |
| Obtains a quality Parasternal Long Axis view | 14 | 14 (100%) | Required |  |  |  |  |
| Points to Right ventricle | 14 | 14 (100%) | Required |  |  |  |  |
| Points to Left ventricle | 14 | 14 (100%) | Required |  |  |  |  |
| Points to Left atrium | 14 | 14 (100%) | Required |  |  |  |  |
| Points to Aortic Valve | 14 | 14 (100%) | Required |  |  |  |  |
| Points to Mitral valve | 14 | 14 (100%) | Required |  |  |  |  |
| Points to Left Ventricular Outflow Track | 14 | 14 (100%) | Required |  |  |  |  |
| Points to Descending thoracic aorta | 14 | 11 (79%) | Vote Again | 13 (93%) | Required |  |  |
| Points to Pericardium | 14 | 14 (100%) | Required |  |  |  |  |
| Selects phased-array transducer | 14 | 14 (100%) | Required |  |  |  |  |
| Selects / uses cardiac exam | 14 | 14 (100%) | Required |  |  |  |  |
| Image depth optimized appropriately | 14 | 14 (100%) | Required |  |  |  |  |
| Image gain optimized appropriately | 14 | 11 (79%) | Vote Again | 13 (93%) | Required |  |  |
| ***Section: POCUS Cardiac Apical Skills Test*** | | | | | | | |
| Correct probe LOCATION? | 14 | 14 (100%) | Required |  |  |  |  |
| Correct probe ORIENTATION? | 14 | 14 (100%) | Required |  |  |  |  |
| Probe CONTROL? | 14 | 14 (100%) | Required |  |  |  |  |
| Speed & EFFICIENCY | 14 | 4 (29%) | No |  |  |  |  |
| Obtains a quality 4- or 5-chamber cardiac view | 14 | 14 (100%) | Required |  |  |  |  |
| Points to Right ventricle | 14 | 14 (100%) | Required |  |  |  |  |
| Points to Left ventricle | 14 | 14 (100%) | Required |  |  |  |  |
| Points to Right atrium | 14 | 14 (100%) | Required |  |  |  |  |
| Points to Left Atrium | 14 | 14 (100%) | Required |  |  |  |  |
| Points to LVOT | 14 | 13 (93%) | Required |  |  |  |  |
| Points to Mitral valve | 14 | 14 (100%) | Required |  |  |  |  |
| Points to Tricuspid valve | 14 | 14 (100%) | Required |  |  |  |  |
| Selects phased-array transducer | 14 | 14 (100%) | Required |  |  |  |  |
| Selects / uses cardiac exam | 14 | 14 (100%) | Required |  |  |  |  |
| Image depth optimized appropriately | 14 | 10 (71%) | Vote Again | 13 (93%) | Required |  |  |
| Image gain optimized appropriately | 14 | 14 (100%) | Required |  |  |  |  |
| ***POCUS Cardiac Subxiphoid Skills Test*** | | | | | | | |
| Correct probe LOCATION? | 14 | 14 (100%) | Required |  |  |  |  |
| Correct probe ORIENTATION? | 14 | 12 (86%) | Required |  |  |  |  |
| Probe CONTROL? | 14 | 13 (93%) | Required |  |  |  |  |
| Speed and Efficiency | 14 | 8 (57%) | No |  |  |  |  |
| Obtains a quality subxiphoid 4-chamber view | 14 | 14 (100%) | Required |  |  |  |  |
| Points to Liver | 14 | 14 (100%) | Required |  |  |  |  |
| Points to Pericardium | 14 | 14 (100%) | Required |  |  |  |  |
| Points to Right Ventricle | 14 | 14 (100%) | Required |  |  |  |  |
| Points to Right Atrium | 14 | 13 (93%) | Required |  |  |  |  |
| Points to Left Ventricle | 14 | 14 (100%) | Required |  |  |  |  |
| Points to Left Atrium | 14 | 11 (79%) | Vote Again | 14 (100%) | Required |  |  |
| Points to Tricuspid Valve | 14 | 11 (79%) | Vote Again | 12 (86%) | Required |  |  |
| Points to Mitral Valve | 14 | 11 (79%) | Vote Again | 12 (86%) | Required |  |  |
| Selected Phased-Array Transducer | 14 | 12 (86%) | Required |  |  |  |  |
| Selects / uses cardiac or abdominal exam preset | 14 | 10 (71%) | Vote Again | 10 (71%) | Vote Again | 13 (93%) | Required |
| 61. Adjusts Image depth optimized appropriately | 14 | 12 (86%) | Required |  |  |  |  |
| Image gain optimized appropriately | 14 | 11 (79%) | Vote Again | 13 (93%) | Required |  |  |
| ***POCUS IVC Skills Test*** | | | | | | | |
| Correct probe LOCATION? | 14 | 14 (100%) | Required |  |  |  |  |
| Correct probe ORIENTATION? | 14 | 11 (79%) | Vote Again | 9 (71%) | Vote Again | 13 (93%) | Required |
| Probe CONTROL? | 14 | 13 (93%) | Required |  |  |  |  |
| Speed & Efficiency | 14 | 8 (57%) | No |  |  |  |  |
| Obtains a quality IVC view | 14 | 14 (100%) | Required |  |  |  |  |
| Points to Liver | 14 | 14 (100%) | Required |  |  |  |  |
| Point to IVC | 14 | 14 (100%) | Required |  |  |  |  |
| Points to Right atrium | 14 | 14 (100%) | Required |  |  |  |  |
| Points to Hepatic Vein | 14 | 12 (86%) | Required |  |  |  |  |
| Point to Site to assess for respiratory variation | 14 | 14 (100%) | Required |  |  |  |  |
| Selected Phased-Array Transducer | 14 | 9 (64%) | Vote Again | 12 (86%) | Required |  |  |
| Selects / uses cardiac or abdominal exam preset | 14 | 10 (71%) | Vote Again | 10 (71%) | Vote Again | 13 (93%) | Required |
| Adjusts Image depth optimized appropriately | 14 | 12 (86%) | Yes |  |  |  |  |
| Image gain optimized appropriately | 14 | 10 (71%) | Vote Again | 13 (93%) | Required |  |  |

| **THORACIC** | | | | | | | | |
| --- | --- | --- | --- | --- | --- | --- | --- | --- |
|  | | **Round 1** | | **Round 2** | | **Round 3** | |  |
| **Proposed Checklist Item** | **# of Voters** | **Required for**  **Competency?** | **Checklist**  **Status** | **Required for**  **Competency?** | **Checklist**  **Status** | **Required for**  **Competency?** | **Checklist**  **Status** |  |
| ***POCUS Thoracic Skills Test*** | |  |  |  |  |  |  |  |
| Correct probe LOCATION? | 14 | 13 (93%) | Required |  |  |  |  |  |
| Correct probe ORIENTATION? | 14 | 11 (79%) | Vote Again | 13 (93%) | Required |  |  |  |
| Probe CONTROL? | 14 | 13 (93%) | Required |  |  |  |  |  |
| Speed and Efficiency? | 14 | 10 (71%) | No |  |  |  |  |  |
| Obtains a quality anterior lung view | 14 | 14 (100%) | Required |  |  |  |  |  |
| Points to Ribs | 14 | 13 (93%) | Required |  |  |  |  |  |
| Points to Rib Shadow | 14 | 13 (93%) | Required |  |  |  |  |  |
| Points to Pleural line | 14 | 14 (100%) | Required |  |  |  |  |  |
| Points to A-lines | 14 | 14 (100%) | Required |  |  |  |  |  |
| Recognizes pleural sliding | 14 | 14 (100%) | Required |  |  |  |  |  |
| Demonstrates normal pattern ("seashore" sign) using M-mode | 14 | 11 (79%) | Vote Again | 13 (93%) | Required |  |  |  |
| Selects linear, phased-array, or curvilinear probe | 14 | 12 (86%) | Required |  |  |  |  |  |
| Selects / uses abdominal or lung exam | 14 | 13 (93%) | Required |  |  |  |  |  |
| Image depth optimized appropriately | 14 | 12 (86%) | Required |  |  |  |  |  |
| Image gain optimized appropriately | 14 | 12 (86%) | Required |  |  |  |  |  |
| ***POCUS Thoracic Costophrenic Skills Test*** | |  |  |  |  |  |  |  |
| Correct probe LOCATION? | 14 | 14 (100%) | Required |  |  |  |  |  |
| Correct probe ORIENTATION? | 14 | 13 (93%) | Required |  |  |  |  |  |
| Probe CONTROL? | 14 | 13 (93%) | Required |  |  |  |  |  |
| Speed and Efficiency? | 14 | 10 (71%) | No |  |  |  |  |  |
| Obtains a quality costophrenic view | 14 | 13 (93%) | Required |  |  |  |  |  |
| Points to Liver or Spleen | 14 | 14 (100%) | Required |  |  |  |  |  |
| Points to Diaphragm | 14 | 14 (100%) | Required |  |  |  |  |  |
| Points to Lung parenchyma | 14 | 14 (100%) | Required |  |  |  |  |  |
| Points to Costophrenic recesses | 14 | 14 (100%) | Required |  |  |  |  |  |
| Selects phased-array or curvilinear probe | 14 | 14 (100%) | Required |  |  |  |  |  |
| Selects / uses abdominal or lung exam | 14 | 12 (86%) | Required |  |  |  |  |  |
| Image depth optimized appropriately | 14 | 11 (79%) | Vote Again | 14 (100%) | Required |  |  |  |
| Image gain optimized appropriately | 14 | 10 (71%) | Vote Again | 13 (93%) | Required |  |  |  |

| **ABDOMINAL** | | | | | | | | |
| --- | --- | --- | --- | --- | --- | --- | --- | --- |
|  |  | **Round 1** | | **Round 2** | | **Round 3** | |  |
| **Proposed Checklist Item** | **# of Voters** | **Required for**  **Competency?** | **Checklist**  **Status** | **Required for**  **Competency?** | **Checklist**  **Status** | **Required for**  **Competency?** | **Checklist**  **Status** |  |
| ***POCUS Abdominal Skills Test*** | | | | | | | |  |
| Correct probe LOCATION? | 14 | 14 (100%) | Required |  |  |  |  |  |
| Correct probe ORIENTATION? | 14 | 13 (93%) | Required |  |  |  |  |  |
| Probe CONTROL? | 14 | 13 (93%) | Required |  |  |  |  |  |
| Speed and Efficiency? | 14 | 9 (64%) | No |  |  |  |  |  |
| Obtains a quality right upper quadrant FAST view | 14 | 14 (100%) | Required |  |  |  |  |  |
| Points to Diaphragm | 14 | 13 (93%) | Required |  |  |  |  |  |
| Points to Liver | 14 | 14 (100%) | Required |  |  |  |  |  |
| Points to Kidney | 14 | 14 (100%) | Required |  |  |  |  |  |
| Points to Morrison’s Pouch | 14 | 14 (100%) | Required |  |  |  |  |  |
| Points to Renal Pelvis | 14 | 14 (100%) | Required |  |  |  |  |  |
| Points to Right paracolic gutter | 14 | 14 (100%) | Required |  |  |  |  |  |
| Selects curvilinear or phased-array probe | 14 | 14 (100%) | Required |  |  |  |  |  |
| Selects / uses abdominal exam | 14 | 13 (93%) | Required |  |  |  |  |  |
| Image depth optimized appropriately | 13 | 12 (93%) | Required |  |  |  |  |  |
| Image gain optimized appropriately | 14 | 12 (86%) | Required |  |  |  |  |  |
| ***POCUS Abdominal / Bladder Skills Test*** | | | | | | | |  |
| Correct probe LOCATION? | 14 | 14 (100%) | Required |  |  |  |  |  |
| Correct probe ORIENTATION? | 13 | 12 (93%) | Required |  |  |  |  |  |
| Probe CONTROL? | 14 | 13 (93%) | Required |  |  |  |  |  |
| Speed and Efficiency? | 14 | 8 (57%) | No |  |  |  |  |  |
| Obtains a quality transverse bladder view. | 14 | 14 (100%) | Required |  |  |  |  |  |
| Points to Bladder wall | 14 | 14 (100%) | Required |  |  |  |  |  |
| Points to Urine within the bladder | 14 | 14 (100%) | Required |  |  |  |  |  |
| Points to Prostate / Uterus | 14 | 12 (86%) | Required |  |  |  |  |  |
| Points to Area to assess for pelvic free fluid | 14 | 14 (100%) | Required |  |  |  |  |  |
| Selects phased-array or curvilinear probe | 14 | 14 (100%) | Required |  |  |  |  |  |
| Selects / uses abdominal exam | 14 | 13 (93%) | Required |  |  |  |  |  |
| Image depth optimized appropriately | 14 | 13 (93%) | Required |  |  |  |  |  |
| Image gain optimized appropriately | 14 | 13 (93%) | Required |  |  |  |  |  |

| **DVT** | | | | | | | | |
| --- | --- | --- | --- | --- | --- | --- | --- | --- |
|  |  | **Round 1** | | **Round 2** | | **Round 3** | |  |
| **Proposed Checklist Item** | **# of Voters** | **Required for**  **Competency?** | **Checklist**  **Status** | **Required for**  **Competency?** | **Checklist**  **Status** | **Required for**  **Competency?** | **Checklist**  **Status** |  |
| ***POCUS Deep Venous Thrombosis - Vascular Skills Test*** | | | | | | | |  |
| *Demonstrates how to acquire quality common femoral vein view:* | | | | | | | |  |
| Correct probe LOCATION? | 14 | 14 (100%) | Required |  |  |  |  |  |
| Correct probe ORIENTATION? | 14 | 13 (93%) | Required |  |  |  |  |  |
| Probe CONTROL? | 14 | 13 (93%) | Required |  |  |  |  |  |
| Speed and Efficiency? | 14 | 8 (57%) | No |  |  |  |  |  |
| Obtains a quality common Femoral Vein view | 14 | 14 (100%) | Required |  |  |  |  |  |
| Points to Common Femoral Vein | 14 | 14 (100%) | Required |  |  |  |  |  |
| Points to Common Femoral/Greater Saphenous Vein Junction | 14 | 14 (100%) | Required |  |  |  |  |  |
| Points to Common Femoral Artery | 14 | 14 (100%) | Required |  |  |  |  |  |
| Demonstrates Common Femoral Vein Compression | 14 | 14 (100%) | Required |  |  |  |  |  |
| Points to Femoral Vein | 14 | 14 (100%) | Required |  |  |  |  |  |
| Demonstrates Femoral Vein Compression | 14 | 14 (100%) | Required |  |  |  |  |  |
| Obtains a quality popliteal vein view | 14 | 14 (100%) | Required |  |  |  |  |  |
| Points to Popliteal Vein | 14 | 14 (100%) | Required |  |  |  |  |  |
| Points to Popliteal Artery | 14 | 14 (100%) | Required |  |  |  |  |  |
| Demonstrates Popliteal Venous Compression | 14 | 14 (100%) | Required |  |  |  |  |  |
| *Demonstrates appropriate operation of portable ultrasound machine to perform a focused deep venous ultrasound exam.* | | | | | | | |  |
| Selects high frequency linear transducer | 14 | 13 (93%) | Required |  |  |  |  |  |
| Selects / uses vascular, venous, or arterial exam | 14 | 12 (86%) | Required |  |  |  |  |  |
| Maintains orientation with probe notch and screen marker | 14 | 12 (86%) | Required |  |  |  |  |  |
| Image depth optimized appropriately | 14 | 13 (93%) | Required |  |  |  |  |  |
| Image gain optimized appropriately | 14 | 11 (79%) | Vote Again | 13 (93%) | Required |  |  |  |

| **PIV** | | | | | | | |
| --- | --- | --- | --- | --- | --- | --- | --- |
|  | | **Round 1** | | **Round 2** | | **Round 3** | |
| **Proposed Checklist Item** | **# of Voters** | **Required for**  **Competency?** | **Checklist**  **Status** | **Required for**  **Competency?** | **Checklist**  **Status** | **Required for**  **Competency?** | **Checklist**  **Status** |
| ***POCUS Peripheral IV Skills Test - Transverse*** | |  |  |  |  |  |  |
| Correct probe LOCATION? | 14 | 12 (86%) | Required |  |  |  |  |
| Correct probe ORIENTATION? | 14 | 12 (86%) | Required |  |  |  |  |
| Probe CONTROL? | 13 | 13 (100%) | Required |  |  |  |  |
| Speed and Efficiency? | 14 | 11 (79%) | No^*^ |  |  |  |  |
| Stabilizes probe to obtain a quality transverse view of vein | 14 | 14 (100%) | Required |  |  |  |  |
| Identifies needle just below skin (probe transverse). | 14 | 14 (100%) | Required |  |  |  |  |
| Tracks needle tip as needle advances toward vein (transverse orientation) | 14 | 14 (100%) | Required |  |  |  |  |
| Successfully uses transverse approach to insert peripheral IV | 14 | 13 (93%) | Required |  |  |  |  |
| Selects high frequency linear transducer | 14 | 14 (100%) | Required |  |  |  |  |
| Selects / uses vascular, venous, or arterial exam | 14 | 12 (86%) | Required |  |  |  |  |
| Maintains orientation with probe notch and screen marker | 14 | 14 (100%) | Required |  |  |  |  |
| Image depth optimized appropriately | 14 | 13 (93%) | Required |  |  |  |  |
| Image gain optimized appropriately | 14 | 10 (71%) | Vote Again | 13 (93%) | Required |  |  |
| ***POCUS Peripheral IV Skills Test - Longitudinal*** | |  |  |  |  |  |  |
| Correct probe LOCATION? | 14 | 12 (86%) | Required |  |  |  |  |
| Correct probe ORIENTATION? | 14 | 12 (86%) | Required |  |  |  |  |
| Probe CONTROL? | 14 | 14 (100%) | Required |  |  |  |  |
| Speed and Efficiency? | 14 | 10 (71%) | No^*^ |  |  |  |  |
| Stabilizes probe to obtain a quality longitudinal view of vein | 14 | 12 (86%) | Required |  |  |  |  |
| Identifies needle just below skin (probe longitudinal). | 14 | 13 (93%) | Required |  |  |  |  |
| Tracks needle tip as needle advances (longitudinal orientation) | 14 | 12 (86%) | Required |  |  |  |  |
| Successfully uses longitudinal approach to insert peripheral IV | 14 | 12 (86%) | Required |  |  |  |  |
| Selects high frequency linear transducer | 14 | 14 (100%) | Required |  |  |  |  |
| Selects / uses vascular, venous, or arterial exam | 14 | 12 (86%) | Required |  |  |  |  |
| Maintains orientation with probe notch and screen marker | 14 | 14 (100%) | Required |  |  |  |  |
| Image depth optimized appropriately | 14 | 12 (86%) | Required |  |  |  |  |
| Image gain optimized appropriately | 14 | 12 (86%) | Required |  |  |  |  |

**After round 1 voting, “Speed and Efficiency” was unanimously decided during group discussion to be removed from all checklists due to inability to standardize this item.*
