# Supplementary material for: Development of a multisystem point of care ultrasound skills assessment checklist
Source: Ultrasound J. 2022 May 12;14:17. doi: 10.1186/s13089-022-00268-4 (PMC9096739; doi:10.1186/s13089-022-00268-4)
Supplement: Supplementary file 3 — Additional file 3: Table S3. Multisystem Point-of-care Ultrasound Checklist for Basic Competency in Image Acquisition and Anatomy Identification. A total of 153 checklist items reached consensus and include four diagnostic applications (cardiac, lung, abdominal, and vascular ultrasound) and one procedural application (peripheral intravenous line insertion). [file 13089_2022_268_MOESM3_ESM.docx]

| **Multisystem POCUS Checklist** |
| --- |
| **Cardiac Parasternal** |
| **Selects PHASED-ARRAY Transducer** |
| **Selects CARDIAC EXAM** |
| **Correct probe LOCATION** (yes/no)  (anterior chest wall, left of mid-sternum, close to sternum; learner may adjust location to optimize view) |
| **Correct probe ORIENTATION** (yes/no)  (probe notch pointed obliquely to right shoulder to obtain an on-axis image) |
| **Probe CONTROL** (adequate/inadequate)   - Adequate = deliberate probe manipulation to fine tune image with hand stabilized on body - Inadequate = gross movements with abrupt changes; sliding all over body |
| **Obtains a quality Parasternal Long Axis view**  Required structures = RV, LV, LA, AV, MV, LVOT, aortic root, descending thoracic aorta   - POOR (new learner) – NO required structures visualized - FAIR (some experience) – SOME required structures visualized - EXCELLENT (experienced) – ALL required structures clearly visualized |
| **Points to RIGHT VENTRICLE** |
| **Points to LEFT VENTRICLE** |
| **Points to LEFT ATRIUM** |
| **Points to AORTIC VALVE** |
| **Points to MITRAL VALVE** |
| **Points to LEFT VENTRICULAR OUTFLOW TRACK** |
| **Points to DESCENDING THORACIC AORTA** |
| **Points to PERICARDIUM** |
| **Image DEPTH optimized appropriately**  (deep enough to see the descending thoracic aorta but not so deep that the entire heart is in the upper half of the screen)   - Too shallow = desired structures extend beyond bottom edge of screen - Too deep = desired structures are seen only in top half of screen |
| **Image GAIN optimized appropriately**  (avoids excessive far field gain)   - Too much gain = excessively BRIGHT image in either or both the near- and far-field - Too little gain = excessively DARK image in either or both the near- and far-field |
| **Cardiac Apical** |
| **Selects PHASED-ARRAY Transducer** |
| **Selects CARDIAC EXAM** |
| **Correct Probe LOCATION** (yes/no)  (centered over point of maximum impulse, typically located in intercostal space 4-5 on the anterolateral chest wall; learner may adjust location to optimize view ) |
| **Correct Probe ORIENTATION** (yes/no)  (probe notch pointed to patient’s left side to obtain an on-axis image) |
| **Probe CONTROL** (adequate/inadequate)   - Adequate = deliberate probe manipulation to fine tune image with hand stabilized on body - Inadequate = gross movements with abrupt changes; sliding all over body |
| **Obtains quality 4-chamber cardiac VIEW** (a 5-chamber view is acceptable)  Required structures = RV, LV, RA, LA, TV, MV   - POOR (New learner) – NO required structures visualized - FAIR (Some experience) – SOME required structures visualized - EXCELLENT (Experienced) – ALL required structures clearly visualized |
| **Points to RIGHT VENTRICLE** |
| **Points to LEFT VENTRICLE** |
| **Points to RIGHT ATRIUM** |
| **Points to LEFT ATRIUM** |
| **Points to MITRAL VALVE** |
| **Points to TRICUSPID VALVE** |
| **Image DEPTH optimized appropriately**  (should be deep enough to see the RA and LA entirely)   - Too shallow = desired structures extend beyond bottom edge of screen - Too deep = desired structures are seen only in top half of screen |
| **Image GAIN optimized appropriately**  (avoids excessively bright near field and excessively dark far field)   - Too much gain = excessively BRIGHT image in either or both the near- and far-field - Too little gain = excessively DARK image in either or both the near- and far-field |
| **Cardiac Subxiphoid** |
| **Selects PHASED-ARRAY Transducer** |
| **Selects CARDIAC EXAM** |
| **Correct Probe LOCATION** (yes/no)  (anterior chest wall, typically just right of midline along right costal margin; learner may adjust location to optimize view) |
| **Correct Probe ORIENTATION** (yes/no)  (probe notch pointed to patient’s left side. Note: the preferred exam preset and orientation can vary based on specialty or local convention) |
| **Probe CONTROL** (adequate/inadequate)   - Adequate = deliberate probe manipulation to fine tune image with hand stabilized on body - Inadequate = gross movements with abrupt changes; sliding all over body |
| **Obtains quality subxiphoid 4-chamber VIEW**  Required structures = Liver, RV, RA, TV, LV, LA, MV   - POOR (New learner) – NO required structures visualized - FAIR (Some experience) – SOME required structures visualized - EXCELLENT (Experienced) – ALL required structures clearly visualized |
| **Points to PERICARDIUM** |
| **Points to LIVER** |
| **Points to RIGHT VENTRICLE** |
| **Points to RIGHT ATRIUM** |
| **Points to LEFT VENTRICLE** |
| **Points to LEFT ATRIUM** |
| **Points to MITRAL VALVE** |
| **Points to TRICUSPID VALVE** |
| **Image DEPTH optimized appropriately**  (deep enough to see all 4 cardiac chambers clearly in the middle portion of the screen)   - Too shallow = desired structures extend beyond bottom edge of screen - Too deep = desired structures are seen only in top half of screen |
| **Image GAIN optimized appropriately**  (avoids excessively dark far field)   - Too much gain = excessively BRIGHT image in either or both the near- and far-field - Too little gain = excessively DARK image in either or both the near- and far-field |
| **IVC Skills** |
| **Selects PHASED-ARRAY or CURVILINEAR Transducer** |
| **Selects CARDIAC or ABDOMINAL EXAM preset** |
| **Correct Probe LOCATION** (yes/no)  (anterior chest wall, typically just right of midline; learner may adjust location to optimize view) |
| **Correct Probe ORIENTATION** (yes/no)  (probe oriented to obtain a long-axis view of the IVC with the probe centered over IVC with the RA visible; IVC is visible across the screen; depending on exam preset and local convention, the probe notch may be pointed cranially or caudally) |
| **Probe CONTROL** (adequate/inadequate)   - Adequate = deliberate probe manipulation to fine tune image with hand stabilized on body - Inadequate = gross movements with abrupt changes; sliding all over body |
| **Obtains quality IVC VIEW**  (IVC should be seen extending across the screen in long axis (i.e., avoid cylinder effect) and is seen connecting to the RA.  Required structures = RA, IVC, a hepatic vein, liver   - POOR (New learner) – NO required structures visualized - FAIR (Some experience) – SOME required structures visualized - EXCELLENT (Experienced) – ALL required structures clearly visualized |
| **Points to LIVER** |
| **Points to IVC** |
| **Points to HEPATIC VEIN** |
| **Points to RIGHT ATRIUM** |
| **Points to Site to assess for RESPIRATORY VARIATION** (~2cm from RA-IVC junction, or just distal to a hepatic vein-IVC junction) |
| **Image DEPTH optimized appropriately**  (IVC should be seen longitudinally across the middle of the screen; avoid excessive depth placing the IVC in the top half of the screen)   - Too shallow = desired structures are seen in the bottom portion of screen - Too deep = desired structures are seen only in top half of screen |
| **Image GAIN optimized appropriately**  (avoids excessively bright far field)   - Too much gain = excessively BRIGHT image in either or both the near- and far-field - Too little gain = excessively DARK image in either or both the near- and far-field |
| **Based on the overall performance of this learner through all the views obtained and identification of anatomic structures during this hands-on skills evaluation, do you consider this learner to have the minimum skills to be considered COMPETENT to be given clinical PRIVILEGES to perform CARDIAC POCUS exams of patients?** |
| **Thoracic Skills** |
| **Selects Phased-Array or Curvilinear Probe** |
| **Selects ABDOMINAL or LUNG EXAM** |
| **Correct Probe LOCATION** (yes/no)  (anterior chest wall in between two ribs, usually with two ribs flanking the image) |
| **Correct Probe ORIENTATION** (yes/no)  [probe notch pointed toward patient’s head (or feet) to give correct image orientation on the screen (screen left = cephalad; screen right = caudad). Probe perpendicular to pleural surface. Must avoid transverse orientation.] |
| **Probe CONTROL** (adequate/inadequate)   - Adequate = deliberate probe manipulation to fine tune image with hand stabilized on body - Inadequate = gross movements with abrupt changes; sliding all over body |
| **Obtains quality Anterior Lung VIEW**  Required structures = Ribs, pleural line, A-lines   - POOR (New learner) – NO required structures visualized - FAIR (Some experience) – SOME required structures visualized - EXCELLENT (Experienced) – ALL required structures clearly visualized |
| **Points to RIBS** |
| **Points to RIB SHADOW** |
| **Points to PLEURAL LINE** |
| **Recognizes PLEURAL SLIDING** |
| **Points to A-LINES** |
| **Demonstrates normal pattern (SEASHORE sign) using M-mode**  (cursor line should be as perpendicular as possible to the pleural line and in between two ribs) |
| **Image DEPTH optimized appropriately**   - Too shallow = not able to visualize A-lines - Too deep = depth >19cm |
| **Image GAIN optimized appropriately**  (avoids excessive gain creating artifacts and noise on the screen)   - Too much gain = excessively BRIGHT image in either or both the near- and far-field - Too little gain = excessively DARK image in either or both the near- and far-field |
| **Thoracic Costophrenic Skills** |
| **Selects Phased-Array or Curvilinear Probe** |
| **Selects ABDOMINAL or LUNG EXAM** |
| **Correct Probe LOCATION** (yes/no)  (lateral chest wall at level of diaphragm between the anterior and posterior axillary lines) |
| **Correct Probe ORIENTATION** (yes/no)  [probe notch pointed toward patient’s head (or feet) to give correct image orientation on the screen (screen left = cephalad; screen right = caudad). Probe perpendicular to pleural surface. May be pointed slightly posteriorly to obtain a clearer image. Must avoid transverse orientation.] |
| **Probe CONTROL** (adequate/inadequate)   - Adequate = deliberate probe manipulation to fine tune image with hand stabilized on body - Inadequate = gross movements with abrupt changes; sliding all over body |
| **Obtains quality Costophrenic VIEW**  Required structures = Diaphragm, sub-diaphragmatic organ (liver or spleen), lung parenchyma descending with respirations (curtain sign)   - POOR (New learner) – NO required structures visualized - FAIR (Some experience) – SOME required structures visualized - EXCELLENT (Experienced) – ALL required structures clearly visualized |
| **Points to LIVER or SPLEEN** |
| **Points to DIAPHRAGM** |
| **Points to LUNG PARENCHYMA** (points to area of “curtain sign” or cephalad of the diaphragm and above the spine on the screen) |
| **Points to COSTOPHRENIC RECESS** |
| **Image DEPTH optimized appropriately**  (avoids excessive depth; can see level of spine with < 2cm of wasted depth in the far-field)   - Too shallow = desired structures extend beyond bottom edge of screen - Too deep = desired structures are seen only in top half of screen |
| **Image GAIN optimized appropriately**  (avoids excessive gain in the far-field)   - Too much gain = excessively BRIGHT image in either or both the near- and far-field - Too little gain = excessively DARK image in either or both the near- and far-field |
| **Based on the overall performance of this learner through all the views obtained and identification of anatomic structures during this hands-on skills evaluation, do you consider this learner to have the minimum skills to be considered COMPETENT to be given clinical PRIVILEGES to perform LUNG & PLEURAL POCUS exams of patients?** |
| **Abdominal Skills** |
| **Selects Curvilinear or Phased-Array Probe** |
| **Selects ABDOMINAL EXAM** |
| **Correct Probe LOCATION** (yes/no)  (lateral abdominal wall in RUQ, below diaphragm in between the anterior and posterior axillary lines) |
| **Correct Probe ORIENTATION** (yes/no)  [probe notch pointed toward patient’s head (or feet) to give correct image orientation on the screen (screen left = cephalad; screen right = caudad). May be pointed slightly posteriorly to obtain a clearer image. Must avoid transverse orientation.] |
| **Probe CONTROL** (adequate/inadequate)   - Adequate = deliberate probe manipulation to fine tune image with hand stabilized on body - Inadequate = gross movements with abrupt changes; sliding all over body |
| **Obtains quality right upper quadrant FAST VIEW**  Required structures = liver, kidney (including inferior pole of kidney), hepatorenal recess   - POOR (New learner) – NO required structures visualized - FAIR (Some experience) – SOME required structures visualized - EXCELLENT (Experienced) – ALL required structures clearly visualized |
| **Points to DIAPHRAGM** |
| **Points to LIVER** |
| **Points to KIDNEY** |
| **Points to HEPATORENAL RECESS (Morison’s Pouch)** |
| **Points to RENAL PELVIS** |
| **Image DEPTH optimized appropriately**  (avoids excessive depth; should see level of spine with < 2cm of wasted depth in the far-field)   - Too shallow = desired structures extend beyond bottom edge of screen - Too deep = desired structures are seen only in top half of screen |
| **Image GAIN optimized appropriately**  (avoids excessive gain in the far-field)   - Too much gain = excessively BRIGHT image in either or both the near- and far-field - Too little gain = excessively DARK image in either or both the near- and far-field |
| **Pelvic Skills** |
| **Selects Curvilinear or Phased-Array Probe** |
| **Selects ABDOMINAL EXAM** |
| **Correct Probe LOCATION** (yes/no)  (anterior abdominal wall in midline over symphysis pubis) |
| **Correct Probe ORIENTATION** (yes/no)  (probe notch pointed to patient’s right side and probe tilted with ultrasound beam aiming into pelvis) |
| **Probe CONTROL** (adequate/inadequate)   - Adequate = deliberate probe manipulation to fine tune image with hand stabilized on body - Inadequate = gross movements with abrupt changes; sliding all over body |
| **Obtains quality transverse BLADDER VIEW with either the prostate or uterus in view**  Required structures = bladder (on-axis view), prostate/uterus   - POOR (New learner) – NO required structures visualized - FAIR (Some experience) – SOME required structures visualized - EXCELLENT (Experienced) – ALL required structures clearly visualized |
| **Points to BLADDER WALL** |
| **Points to PROSTATE or UTERUS** |
| **Points to URINE WITHIN THE BLADDER** |
| **Points to area to assess for PELVIC FREE FLUID**   1. Men= points to rectovesicular space (posterior to bladder and prostate) 2. Women= points to rectouterine space (posterior to bladder and uterus) |
| **Image DEPTH optimized appropriately**  (should be deep enough to see free fluid posterior and inferior to bladder)   - Too shallow = desired structures extend beyond bottom edge of screen - Too deep = desired structures are seen only in top half of screen |
| **Image GAIN optimized appropriately**  (far-field should not appear excessively bright obscuring view of prostate/uterus and rectum)   - Too much gain = excessively BRIGHT image in either or both the near- and far-field - Too little gain = excessively DARK image in either or both the near- and far-field |
| **Based on the overall performance of this learner through all the views obtained and identification of anatomic structures during this hands-on skills evaluation, do you consider this learner to have the minimum skills to be considered COMPETENT to be given clinical PRIVILEGES to perform ABDOMINAL & PELVIC POCUS exams of patients?** |
| **DVT - Vascular Skills** |
| **Selects High Freq Linear Transducer** |
| **Selects Vascular, Venous, or Arterial EXAM** |
| **Correct Probe LOCATION** (yes/no)  (starts on proximal thigh in inguinal crease) |
| **Correct Probe ORIENTATION** (yes/no)  (probe notch pointed to patient’s right side to give correct image orientation on the screen. Probe held perpendicular to the skin surface of the thigh and not tilted) |
| **Probe CONTROL** (adequate/inadequate)   - Adequate = deliberate probe manipulation to fine tune image with hand stabilized on body - Inadequate = gross movements with abrupt changes; sliding all over body |
| **Obtains quality COMMON FEMORAL VEIN VIEW** (yes/no)  (vein should appear oval/triangular clearly in the center of screen; avoid oblique, off-axis views) |
| **Points to common femoral vein** |
| **Points to common femoral artery** |
| **Points to common femoral vein/greater saphenous vein junction** |
| **Demonstrates common femoral vein COMPRESSION**  (short, brisk compressions while maintaining vein centered on screen. Compresses the vein until fully collapsed or the nearby artery deforms) |
| **Points to femoral vein** |
| **Demonstrates femoral vein COMPRESSION**  (short, brisk compressions while maintaining vein centered on screen. Compresses the vein until fully collapsed or the nearby artery deforms) |
| **Obtains quality POPLITEAL VEIN VIEW** (yes/no)  (both popliteal vein and artery should be seen clearly in center of screen) |
| **Points to popliteal artery** |
| **Points to popliteal vein** |
| **Demonstrates popliteal venous COMPRESSION**  (short, brisk compressions while maintaining vein centered on screen. Compresses the vein until fully collapsed or the nearby artery deforms) |
| **Image DEPTH optimized appropriately**  (target vessels should be in the center of the screen)   - Too shallow = target vessels seen in the bottom 1/3 of screen or extend beyond bottom edge of screen - Too deep = target vessels seen in the top 1/3 of screen |
| **Image GAIN optimized appropriately**  (avoids excessively bright far field)   - Too much gain = excessively BRIGHT image in either or both the near- and far-field - Too little gain = excessively DARK image in either or both the near- and far-field |
| **Based on the overall performance of this learner through all the views obtained and identification of anatomic structures during this hands-on skills evaluation, do you consider this learner to have the minimum skills to be considered COMPETENT to be given clinical PRIVILEGES to perform LOWER EXTREMITY DVT POCUS exams of patients?** |
| **Peripheral IV Skills TRANSVERSE APPROACH** |
| **Selects High Freq Linear Transducer** |
| **Select Vascular, Venous, or Arterial EXAM** |
| **Correct Probe LOCATION** (yes/no)  (circular vessel is seen in transverse plane in center of screen; avoid oblique, off-axis views) |
| **Correct Probe ORIENTATION** (yes/no)  (probe notch should be pointed toward operator’s left side) |
| **Probe CONTROL** (adequate/inadequate)   - Adequate = deliberate probe manipulation to maintain vessel in center of screen with hand stabilized on body - Inadequate = gross movements with abrupt changes; sliding all over body |
| **Tracks needle tip as needle advances toward vein** (transverse orientation) (learner must slowly and deliberately track the needle tip; avoids serial attempts with redirections; needle advancements and withdrawals should be deliberate without guessing) |
| **Successfully uses transverse approach to insert peripheral IV** |
| **Image DEPTH optimized appropriately**  (target vessel should be in the center of the screen)   - Too shallow = target vessel seen in the bottom 1/3 of screen or extends beyond bottom edge of screen - Too deep = target vessel seen in the top 1/3 of screen |
| **Image GAIN optimized appropriately**  (avoids excessively bright far field)   - Too much gain = excessively BRIGHT image in either or both the near- and far-field - Too little gain = excessively DARK image in either or both the near- and far-field |
| **Peripheral IV Skills LONGITUDINAL** |
| **Selects High Freq Linear Transducer** |
| **Selects Vascular, Venous, or Arterial EXAM** |
| **Correct Probe LOCATION** (yes/no)  (vessel is seen longitudinally across screen without tapering; avoid oblique, off-axis views) |
| **Correct Probe ORIENTATION** (yes/no)  (probe notch pointed toward operator; needle should be seen traveling from left to right on screen) |
| **Probe CONTROL** (adequate/inadequate)   - Adequate = deliberate probe manipulation to maintain longitudinal view of vein with hand stabilized on body - Inadequate = avoids sliding off vessel; avoids gross movements with abrupt changes |
| **Tracks needle tip as needle advances toward target vessel in longitudinal orientation** (maintains needle within plane of ultrasound beam and keeps entire needle in sight while advancing ) |
| **Successfully uses longitudinal approach to insert peripheral IV** |
| **Image DEPTH optimized appropriately**  (target vessel should be in the center of the screen)   - Too shallow = target vessel seen in the bottom 1/3 of screen or extends beyond bottom edge of screen - Too deep = target vessel seen in the top 1/3 of screen |
| **Image GAIN optimized appropriately**  (avoids excessively bright far field)   - Too much gain = excessively BRIGHT image in either or both the near- and far-field - Too little gain = excessively DARK image in either or both the near- and far-field |
| **Based on the overall performance of this learner through all the views obtained and identification of anatomic structures during this hands-on skills evaluation, do you consider this learner to have the minimum skills to be considered COMPETENT to be given clinical PRIVILEGES to use POCUS for PERIPHERAL IV INSERTION on patients?** |
